# Conception and bicentric validation of the proSCANNED score, a simplified bedside prognostic score for Heart Failure patients

**DOI:** 10.1038/s41598-021-85767-5

**Published:** 2021-03-17

**Authors:** Claire Duflos, Kamila Solecki, Michel Galinier, Romain Itier, Jerome Roncalli, Anne-Laure Goger, Bara Mandoraah, Eran Kalmanovich, Fabien Huet, Audrey Agullo, Delphine Delseny, Jean-Christophe Macia, Florence Leclercq, Gregoire Mercier, François Roubille

**Affiliations:** 1 Medical Department Information, CHU Montpellier, Univ Montpellier, Montpellier, France; 2 IDESP, Univ Montpellier, INSERM, Montpellier, France; 3 Department of Cardiology, Montpellier University Hospital, CHRU Arnaud de Villeneuve, 371 avenue du Doyen Gaston Giraud, 34295 Montpellier Cedex 5, France; 4 Department of Cardiology, University Hospital of Rangueil, 1 avenue Professeur Jean Poulhès, TSA 50032, 31059 Toulouse Cedex, France; 5 UMR UT3 CNRE 5288 Evolutionary Medicine, Obesity and Heart Failure: Molecular and Clinical Investigations. INI-CRCT F-CRIN, GREAT Networks, Toulouse Cedex, France; 6 Département de Cardiologie, Hôpital Arnaud de Villeneuve, CHU de Montpellier, UFR de Médecine, Université Montpellier 1, 371, avenue du Doyen Gaston Giraud, 34295 Montpellier cedex 05, France

**Keywords:** Cardiology, Prognosis

## Abstract

A simple and accurate prognostic tool for Heart Failure (HF) patients is critical to improve follow-up. Different risk scores are accurate but with limited clinical applicability. The current study aims to derive and validate a simple predictive tool for HF prognosis. French outpatients with stable HF of two university hospitals were included in the derivation (N = 134) or in the validation (N = 274) sample and followed up for a median of 23 months. Potential predictors were variables with known association with mortality and easily available. The proSCANNED risk score was derived using a parametric survival model on complete case data; it includes 8 binary variables and its values are 0–8. In the validation sample, the ability of the score to discriminate the 1-year vital status was moderate (AUC = 0.71, IC95% = [0.64–0.71]). However, the stratification of the score in three groups showed a good calibration for patients in the low- and medium-risk risk group. The proSCANNED score is an easy-to-use tool in clinical practice with a good discrimination, stability, and calibration sufficient to improve the medical care of patients. Other follow up studies are necessary to assess score applicability in larger populations, and its impact.

## Introduction

Heart failure (HF) is a major public health concern and cause of death and hospitalization^1,2^. The increasing HF incidence in the population explains the growing need for new tools to enable accurate prognostic assessment. This will allow for propper treatment and monitoring, thereby improving patients’ quality of life and reducing the cost burden on the health system.

Different risk models for patients with HF have already been developed^3^. Most of the models are based on single cohorts of patients and some are restricted to patients with reduced left ventricular ejection fraction^4–6^ while other models are more complex, which limits their applicabillity to daily practice. The Meta-Analysis Global Group in Chronic Heart Failure (MAGGIC)^7^ was based on the largest available database of HF patients- 39,372 patients from 30 studies, with a median follow-up of 2.5 years and aimed to provide a user-friendly score to quantify patient mortality risk. This score was built from 13 routinely available patient characteristics and can be calculated using an online calculator. Importantly, this prediction model had an external validation by the Swedish Heart Failure Registry population^8^.

Although the MAGGIC score is supposed to be user-friendly, it is actually difficult to apply in practice for patients during outpatient consultation or at bedside visits because there are more than 10 characteristics to implement and the use of electronic devices is mandatory. By contrast, the CHADS score and later versions^9^ became a corner stone for the management of the patients due to their simplicity to use. A score available for the management of patients with HF could be valuable to guide treatment of follow-up.

Our aim was to define and validate a user-friendly, easily calculated score without using any device or website, based on the methodology and the characteristics defined by the MAGGIC score.

## Material and methods

### Population

Three clinical databases were used for this study. One database was used to derive the score (derivation sample); the two other databases were pooled and used for the validation (validation sample). The derivation sample included patients with stable HF who attended outpatient clinic visits in the University Hospital of Montpellier (CHU Montpellier, France) between May 2010 and February 2011. The validation sample included patients diagnosed with acute or chronic HF at least 6 months prior to study initiation, as recommended by the European Society of Cardiology^10^, and attending outpatient clinic visits in 2012 in the CHU Montpellier and in the University Hospital of Toulouse (France). Inclusion criteria were being older than 18 years of age and having confirmed HF diagnosis. Exclusion criteria were suffering from acute coronary syndrome within 1 month prior to induction into the study, cardiac surgery, and chemotherapy. HF etiology and treatment type had no influence on patient inclusion or exclusion.

The protocol was performed according to the principles of the Declaration of Helsinki, approved by the International Review Board of Montpellier University Hospital (198711). Following the European and French reglementation, informed consent was obtained from all patients (only adults were included). No patient stated his/her opposition to authors.

### Potential predictors assessment

Comorbidities such as hypertension, diabetes, dyslipidemia, body mass index (BMI, kg/m^2^), atrial fibrillation (AF) (regardless of its characteristic – paroxystic or permanent), chronic obstructive pulmonary disease (COPD), smoking habits, were recorded. Other clinical variables such as age, gender, New York Heart association (NYHA) class, ischemic cardiomyopathy etiology, left ventricular ejection fraction (LVEF), presence of implantable cardioverter-defibrillator (ICD), HF medications were recorded. All these variables were prospectively collected by research teams, before the assessment of the outcome.

At each visit in cardiology wards, results of recent biological exams are checked (< 7 days); if no recent exam exists, they are completed during the visit. Nt-proBNP and Creatinine were collected in these exams. Nt-pro-Brain Natriuretic Peptide (Nt-pro-BNP, pg/mL) was determined using an immuno-electrochemiluminescence assay on the Cobas8000/e6021 immunochemistry system (Roche Diagnostics, Meylan, France). Renal function was assessed with creatinemia (μmol/L) performed on Cobas 8000/c7011 and ISE (Roche, Meylan, France). Glomular filtration rate (GFR) was estimated by Chronic Kidney Disease Epidemiology Collaboration (GFR CKD-EPI) equation.

### Choice of the variables to be tested in the predictive model

The original mortality model of the MAGGIC project had a high statistical power, and was validated in an external sample^8^. Therefore, we used the variables with the most significant prediction power from the MAGGIC model and discarded those with low prediction. Variables that were time consuming to collect or that were subject to inaccuracies (i.e. lowest systolic blood pressure (SBP), HF duration) were excluded. Interactions between variables (“interaction of ejection fraction and age” and “interaction of ejection freaction and SBP”) were also excluded. These exclusions were as such in order to establish a more “user friendly” score. Natriuretic peptides were included as potential predictors due to their increasing availability and significance as a gold-standard predictive marker of HF^10,11^.

The linearity of the link between quantitative predictors and time to death was assessed in univariate Cox models following validation of the proportional hazards hypothesis. When no linear link was found variables were dichotomized using thresholds that were clinically suggestive and statistically effective. The logarithm of the Nt-proBNP was used for model fitting, but results are presented using real values.

### Outcome measure

The primary outcome was one-year mortality. The secondary outcome was mortality during the whole follow-up. Data collection was performed by analyzing the medical files and by phone with the cardiologist or general practitioner, the patient or the family. No blinding of outcome assessment was performed.

### Statistical methods

#### Sample size

All patients included in the prospective database and meeting the inclusion criteria were included.

#### Model derivation

Poisson regression models were used to simultaneously relate baseline variables to the time to death from any cause. Since mortality risk is higher early on, the underlying Poisson rate was set in three time periods: up to 1 year, 1 to 3 years, and over 3 years. These time periods were chosen because the probability of dying within 1 year is an important prognostic feature, which helps to assess the risk–benefit balance of invasive therapeutic procedures. The model was built using backward stepwise regression targeting the minimal AIC. Missing values were not imputed.

#### Mortality risk score

The Poisson model predictor was converted to an integer score, which is then directly related to an individual’s probability of dying within 4.4 years (the longest follow-up). Each integer is a rounding of the exact coefficient in the Poisson model, making log rate ratio 1 equivalent to 1 point. A zero score represents a patient at lowest possible risk. Having only binary variables, the score increases by an integer amount for each risk factor. This method allows computing a predicted mortality risk at 1 year and a predicted mortality risk at the longest follow-up. We plotted the ROC curve of the score, and presented the area under the receiver operating characteristic (ROC) curve (AUC) as an overall measure of model discrimination. The 95% confidence interval of this AUC was calculated by a bootstrap method.

#### Validation of the score

An external validation on the validation sample was performed. The scores and predicted probabilities of death were computed. The ROC curve of the score was plotted and the AUC represents, an overall measure of model discrimination. The 95% confidence interval of this AUC was calculated by a bootstrap method. In order to assess the calibration of the score at one year, patients lost to follow-up before one year were excluded. As an overall summary measure of calibration, the observed 1-year mortality compared with the mean 1-year predicted mortality ratio was calculated.

## Results

### Population

173 patients were included in the derivation sample (Table 1). Of these patients only 134 had complete data and were included in the final analysis. The mean patient age was 75 year, 31% of whom were female. Most of the patients (79, 46%) presented with dyspnea graded as NYHA class III. LVEF ≤ 35% was observed in 57% of patients. Other comorbidities included hypertension in 63% of patients, 36% were diabetics, 47% had dyslipidemia, and 12% had a history of atrial fibrillation. The most common cardiomyopathy was coronary artery disease (49%). Not all patients were treated with long-term treatments (Table 1), and patients taking these medications had not all the maximal dose: mean dose of beta-blockers, ACE-I/ARB, and MRA, represented respectively 38%, 36%, and 51% of maximal dose. Overall, 65 (38%) patients died during a median follow-up of 42 months (3.5 years). Median follow-up was of 42 months (min 0 days, max 4.4 years). Table 1 further describes the derivation sample.

**Table 1:** Description of samples.

	Derivation sample				Validation sample	
	Total (N = 173)	Alive (N = 108)	Dead (N = 65)	p*	(N = 274)	p**
Age, years	75 (66–81)	72 (63–79)	79 (72–84)	0.002	64 (52–72)	< 0.001
Male	119 (69)	72 (67)	47 (72)	0.54	201 (63)	0.29
LVEF ≤ 35%	98 (57)	59 (55)	39 (60)	0.56	177 (65)	0.09
**NYHA**						
I	10 (6)	9 (8)	1 (2)	0.01	20 (7)	< 0.001
II	53 (31)	39 (36)	14 (22)		135 (50)	
III	79 (46)	46 (43)	33 (51)		96 (35)	
IV	31 (18)	14 (13)	17 (26)		23 (8)	
**Comorbidities**						
BMI, kg/m^2^	26 (23–30)	26 (22–30)	26 (23–29)	0.91	26 (23–28)	0.17
Hypertension	109 (63)	61 (56)	48 (74)	0.07	104 (40)	< 0.001
Dyslidemia	81 (47)	49 (45)	32 (49)	0.73	111 (41)	0.19
Current smoker	82 (47)	54 (50)	28 (43)	0.53	50 (18)	< 0.001
Diabetes	62 (36)	29 (27)	33 (51)	0.002	58 (21)	< 0.001
AF	12 (7)	8 (7)	4 (6)	1	0(0)	1
Ischaemic cardiomyopathy	84 (49)	51 (47)	33 (51)	0.51	129 (47)	0.76
COPD	39 (23)	23 (21)	16 (25)	0.73	41 (15)	0.04
**Biochemical analysis**						
Nt-proBNP, pg/mL	2347 (814–5607)	1812 (565–3535)	3687 (1679–11,000)	< 0.001	1851 (690–4750)	0.15
Creatinine, μmol/L	102 (83–137)	93 (79–125)	117 (93–146)	0.004	106 (87–130)	0.83
GFR CKD-EPI, mL/min/1.73m^2^	54 (38–75)	62 (45–84)	49 (31–65)	0.002	62 (43–78)	0.002
**Medications**						
Beta-blockers	118 (68)	84 (78)	34 (52)	0.02	206 (75)	0.11
ACE-I/ARB	119 (69)	82 (76)	37 (57)	0.02	187 (68)	0.95
MRA	54 (31)	44 (41)	10 (15)	0.002	132 (48)	< 0.001
Digitalis	8 (5)	2 (2)	6 (9)	0.05	0 (0)	0.05
Ivabradine	7 (4)	3 (3)	4 (6)	0.31	33 (12)	0.004
ICD	51 (29)	34 (31)	17 (26)	0.02	95 (35)	0.25
CRT	28 (16)	19 (18)	9 (14)	0.84	52 (19)	0.07

Values are median (1st and 3rd quartiles) or N (%).

*ACE-I* angiotensin converting enzyme inhibitor; *AF* atrial fibrillation; *ARBs* angiotensin receptor antagonist; *BMI* body mass index; *CKD-EPI* Chronic Kidney Disease Epidemiology Collaboration; *COPD* chronic obstructive pulmonary disease; *LVEF* left ventricular ejection fraction; *GFR* glomerular filtration rate; *ICD* implantable cardioverter-defibrillator; *CRT* cardiac resynchronization therapy; *MRA* Mineralocorticoid Receptor Antagonists; *Nt-proBNP* Nt-pro brain natriuretic peptide; *NYHA* New York Heart Association; *p* p-value.

*Comparison of alive and dead patients from the derivation sample.

**Comparison of the derivation and the validation sample.

274 patients were included in the validation sample, with 41 death (15%). Comparatively to the derivation sample, patients of the validation sample were younger, with less comorbidities and a better NYHA status (Table 1). Median follow-up was of 23 months (min 9 days, max 4.2 years).

### Model derivation

There were 11 baseline variables available for inclusion in our prognostic model. Table 1 provides their descriptive statistics and univariate analysis. As missing data could not be imputed, we derived the multivariate model on 134 patients. Using Poisson regression models for patient survival with backward stepwise variable selection, adjusting for follow-up time (higher mortality rate in early follow-up), 8 independent predictor variables were identified (Table 2).

**Table 2:** Baseline variables considered for inclusion in the model.

	Alive % (n = 108)	Died % (n = 65)	Missing values %	p-value (Log-rank)
Age ≥ 75, years	39	69	0	< 10^–4^
BMI < 25, kg/m^2^	44	43	0	0.74
LVEF ≤ 35%	25	31	2	0.15
Creatinine ≥ 83, μmol/L	67	85	0	0.01
NT-proBNP > 5000, pg/mL	17	46	0	< 10^–5^
NYHA III-IV	56	77	0	0.004
Male	67	72	0	0.42
Diabetes	27	51	17	< 10^–3^
COPD	21	25	3	0.44
Current smoker	50	43	14	0.84
No beta-blockers	24	31	9	0.13

*BMI* body mass index; *COPD* chronic obstructive pulmonary disease; *LVEF* left ventricular ejection fraction; *Nt-proBNP* Nt-pro brain natriuretic peptide; *NYHA* New York Heart Association.

### Mortality risk score

**Figure 1:**
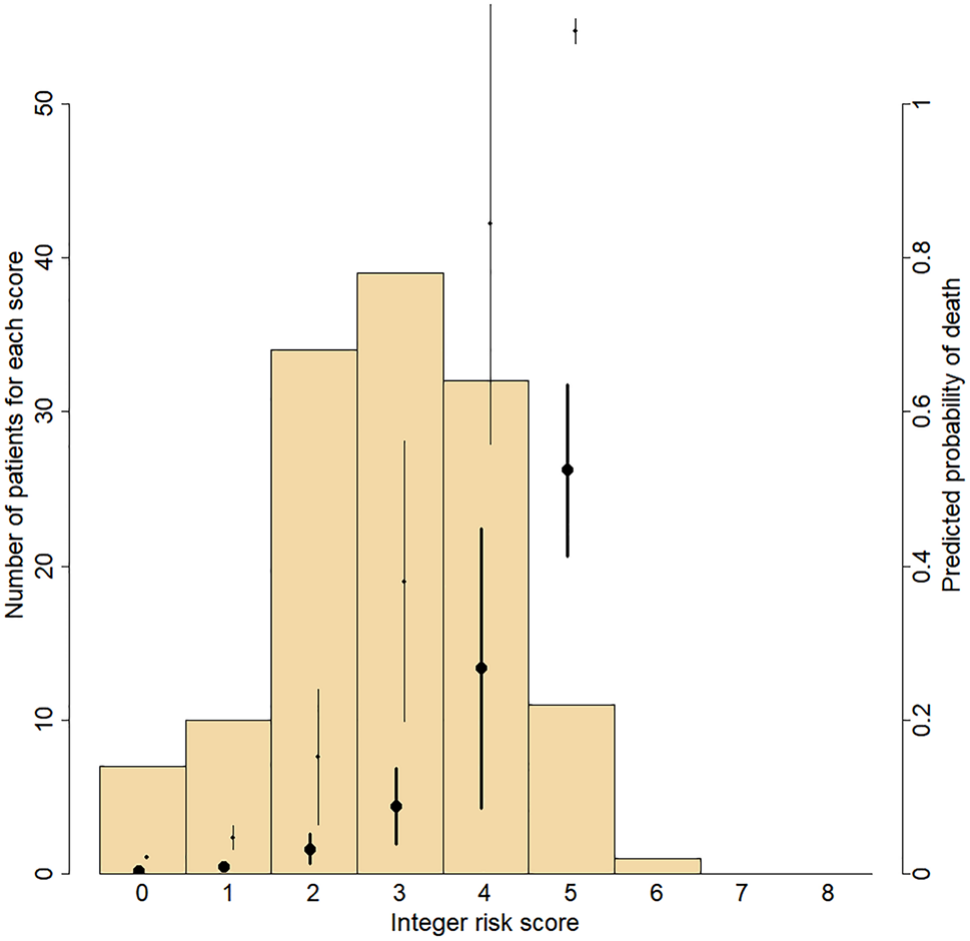
Integer risk score and probability of dying within 1 and 4.4 years in the derivation sample. The histogram shows the number of patients for each score. The points and error bars show the mean predicted probabilities and their Wald’s 95% Confidence Interval. In bold, the predicted probabilities of dying within 1 year; in light, the predicted probabilities of dying within 4.4 years.

From the risk coefficients given in Table 3, an integer score has been created. One point was attributed to each risk factor. The bell-shaped distribution of this integer risk score for all 134 patients is shown in Fig. 1. The points relate a patient’s score to his probability of dying within 1 and 4.4 years. For instance, scores of 2, 3, and 4 have 1-year probabilities of 0.05, 0.13, and 0.32, respectively. The values for scores of 6 and 7 are not represented, because they exceed the graphic zone.

**Table 3:** Multivariate analysis.

	Rate ratio	95% CI	Log rate ratio	p-value
Age ≥ 75 years	3.01	(1.61–5.63)	1.10	0.0006
NYHA III-IV	2.28	(1.12–4.65)	0.83	0.0228
LVEF ≤ 35%	2.61	(1.31–5.17)	0.96	0.0061
Nt-proBNP > 5000, pg/mL	3.22	(1.64–6.33)	1.17	0.0007
No betablockers	3.55	(1.70–7.42)	1.27	0.0007
Diabetes	3.32	(1.74–6.34)	1.20	0.0003
COPD	1.79	(0.84–3.82)	0.58	0.1340
Current smoker	1.83	(0.92–3.64)	0.61	0.0844

Piecewise constant exponential model.

*CI* confidence interval; *COPD* chronic obstructive pulmonary disease; *LVEF* left-ventricular ejection fraction; *Nt-proBNP* Nt-pro brain natriuretic peptide; *NYHA* New York Heart Association.

**Figure 2:**
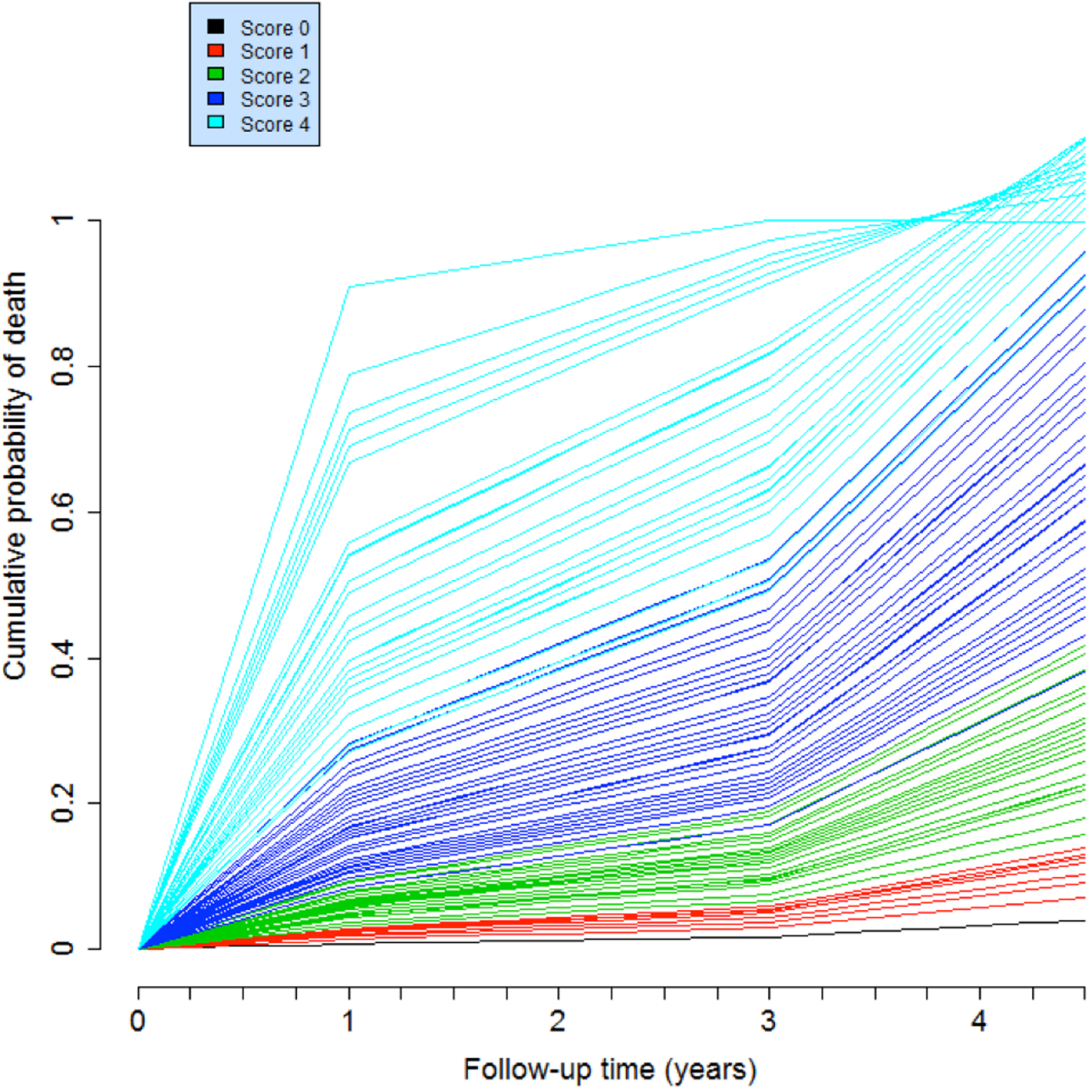
Predicted mortality over 4.4 years for theoretical patients in the scores 0 to 4. Cumulative mortality risk over 4.4 years for theoretical patients with scores of 0 to 4. Scores ≥ 5 were not represented because they need larger scale of the vertical axis, limiting analysis of lower scores.

**Figure 3:**
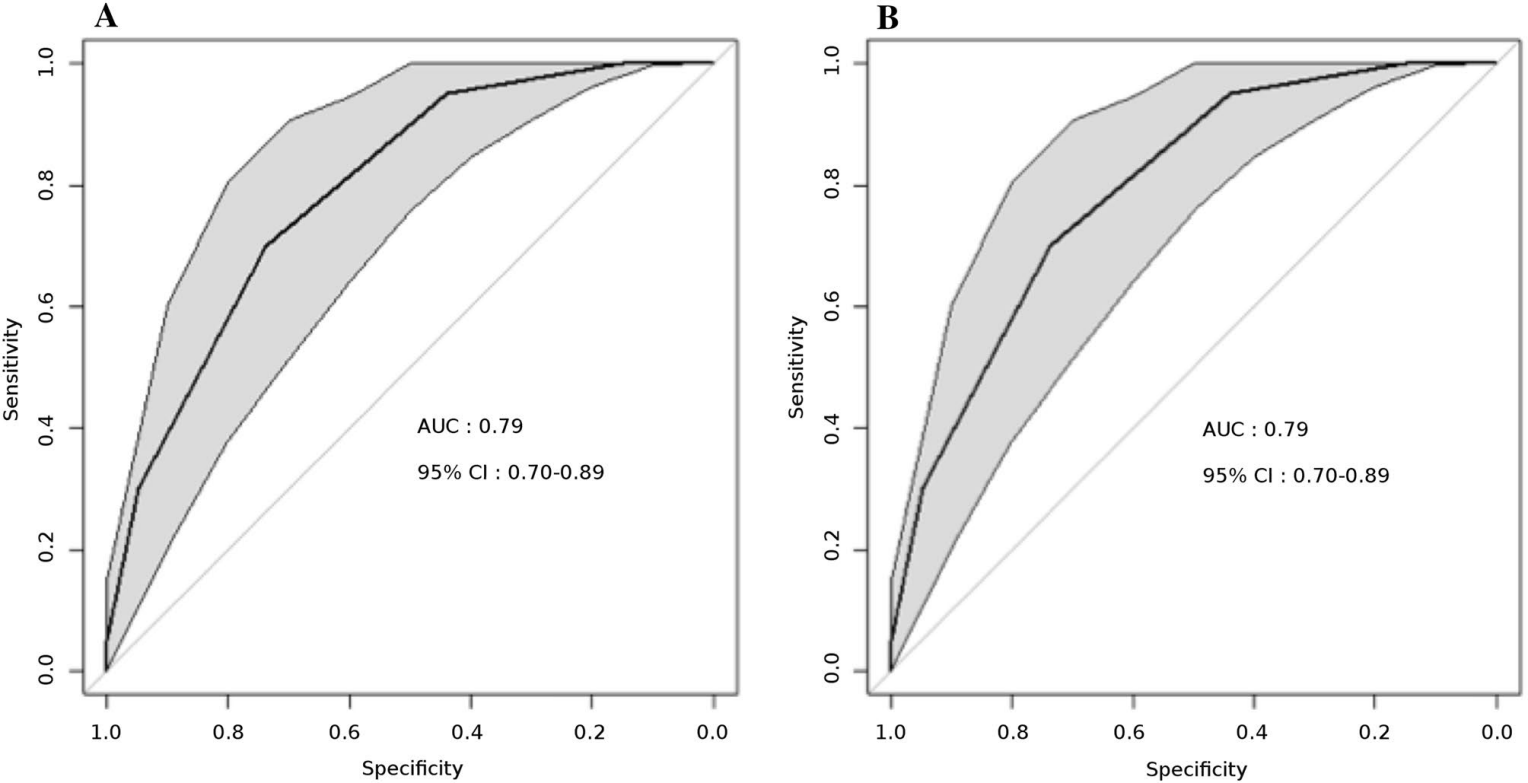
ROC curves of the integer score for the prediction of 1 and 4.4-years mortality in the derivation sample. (**A**) 1 year mortality. (**B**) 4.4 years mortality. The grey shape displays the 95% confidence interval of sensitivity. ROC receiver operating characteristics. AUC area under the curve. CI confidence interval. CI were computed using a boot-strap method.

Figure 2 shows predicted mortality over 4.4 years for theoretical patients in the scores 0 to 5. One curve is drawn for each possible combination of risk factors in each score. For example, there are eight possibilities to have a score at 1, so there are eight curves. The ROC curves of the integer score for the prediction of 1 and 4.4-years mortality are displayed in Fig. 3.

### Validation of the mortality risk score

**Figure 4:**
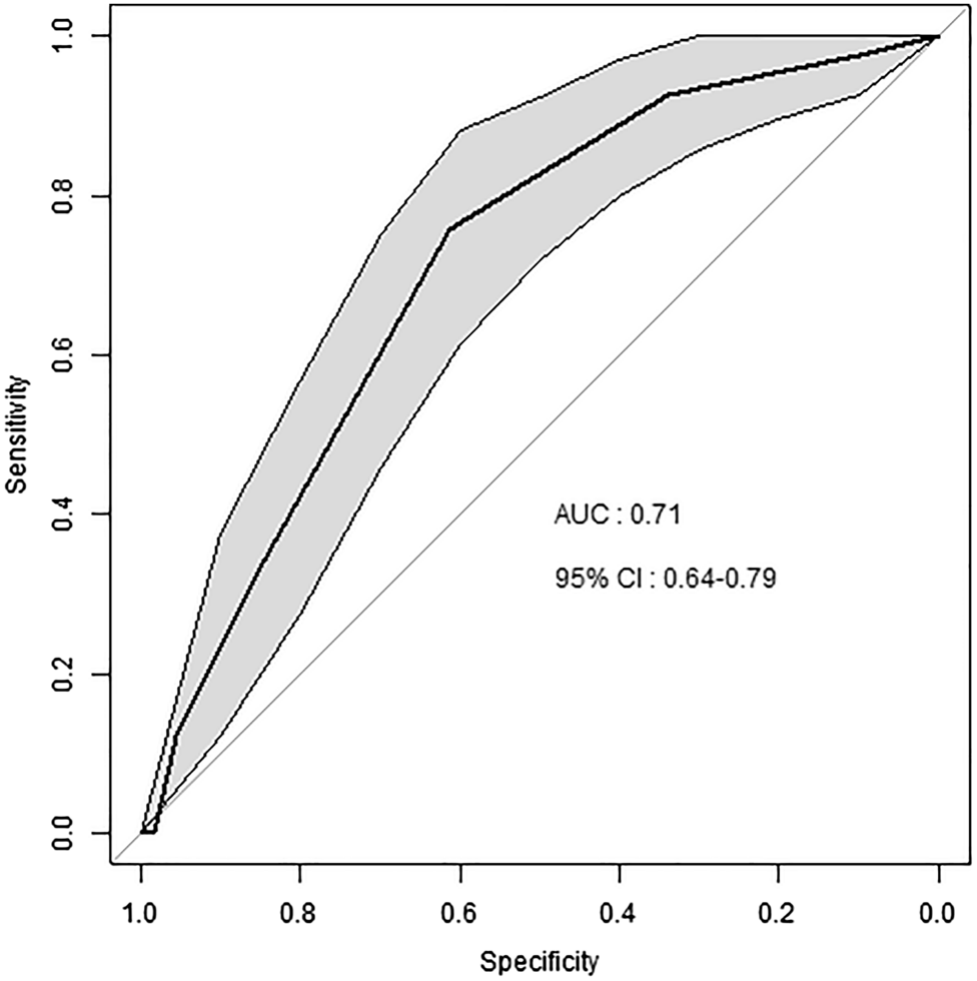
ROC curves of the integer score for the prediction of 1 and 4.4-years mortality in the validation sample. The grey shape displays the 95% confidence interval of sensitivity. *ROC* receiver operating characteristics. *AUC* area under the curve. *CI* confidence interval. CI were computed using a boot-strap method.

**Figure 5:**
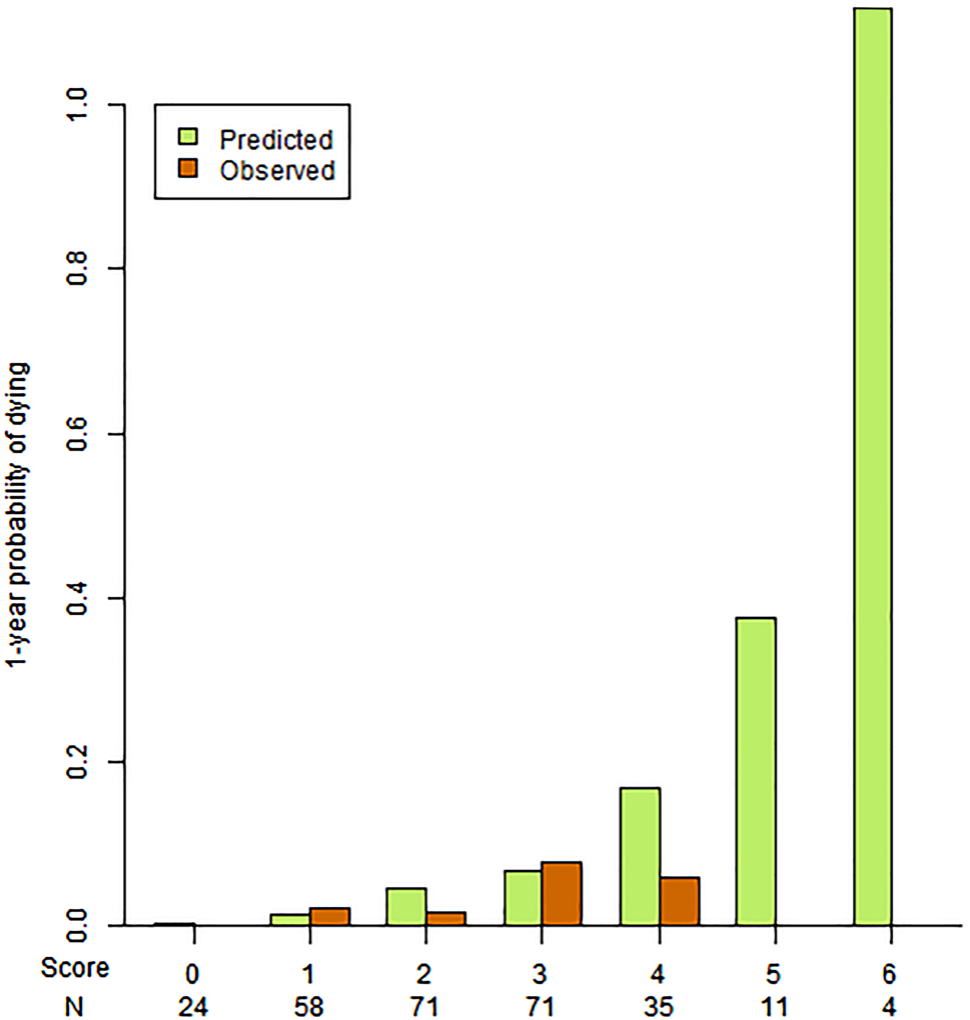
Calibration of the score in the validation sample, for the mortality risk at 1 year.

The mortality risk score was computed on 226 patients (82%) of the validation sample, due to missing values. The discrimination was good: the AUC was of 0.71 (IC 95% 0.64–0.79) (Fig. 4). The calibration is represented in Fig. 5.

## Discussion

### Main results

Similarly to the CHADS_2_ score for atrial fibrillation, we propose here a simple score easy to evaluate, in order to help clinicians to tackle heart failure. Indeed, in patients with higher score, the management has to be reinforced (drugs, rehabilitation, etc.) and patients’ education has to be implemented in these patients being in life-threatening condition. In the present study, 8 independent predictors of mortality in HF were identified. All these factors have been already described and proven to play a main role in the determination of the HF prognosis, and are routinely available in the medical history.

We based our scoring system on the original mortality model of MAGGIC^7^ which had a high statistical power. Comparing to the MAGGIC project our score seems to be by far easier for clinicians to use in their daily practice for several reasons: 1/we propose fewer predictors (our 8 comparing to MAGGIC’s) 2/they are easily obtained, evidenced by our low rate of missing data 3/each predictor is considered equivalent and provides 1 point which in turn can easily be calculated without the need for electronic device and thus save a valuable time for the physician. 4/Utilization of acronym “proSCANNED” (“pro” for “Nt-proBNP > 5000 pg/mL”, “S” for “smoking”, “C” for COPD, “A” for “age ≥ 75 years-old”, double N: one for “No betablockers”, the second one for “ NYHA III or IV class dyspnea”, “E” for “ejection fraction ≤ 35%”, “D” for “diabetes”) can be used to aid in the application of this score, with each item providing one point. 5/ The integer risk of proSCANNED provides an evaluation of the one-year mortality, more relevant than 3-year mortality rate studied by MAGGIC. One-year mortality appears for clinicians in practice as a critical time span not only to help them to adapt the frequency of medical consultations and to promote medical therapies increase but also to assess the risk–benefit balance for invasive therapies such as implantable cardioverter defibrillators, left ventricular assist devices or heart transplantation. In addition, especially in an elderly population, risk stratification should be repeated after one year because comorbidities may change but at the same time other predictors might also change^12^. Importantly, the only biological marker assessed in the score is a natriuretic peptide usually available at least once a year.

Of our selected predictors, four of them (age, NYHA class, ejection fraction and diabetes) had been previously identified by the MAGGIC model and described in other scores as well^13,14^. The other disease indicator of a poorer prognosis was prevalence of COPD, identified as an underdiagnosed HF comorbidity^10^. The most important factor in our study seems to be “no beta-blockers” with the highest rate ratio (3.55, 95% CI 1.70–7.42) already highly discussed in literature^15^. In regard to LVEF, it is well established that HF patients with reduced ejection fraction, in particular if below 40%, have a worse prognosis than with preserved ejection fraction^7,13^. In HF-ACTION, ambulatory patients with HF with systolic dysfunction (LVEF ≤ 35%) have a high rate of morbidity and mortality despite widespread use of evidence-based therapies^5^. Nevertheless, absolute mortality remains high in HF patients with preserved HF, thus highlighting the need to identify others prognostic factors.

The adjunction of natriuretic peptides appears crucial because they are currently recognized as gold-standard predictive markers in HF, and were already incorporate in prognostic scores of HF^4,14^. During HF, there is a myocardial stress resulting in neurohormonal activation by natriuretic peptides. One advantage of our score could be to underline the interest of beta-blockers each time the clinician calculates the score. Natriuretic peptides are recommended by 2013 ACC/AHA guidelines^11^ and 2016 ESC guidelines^10^ for diagnosis and prognosis in chronic HF (class I) and should be used with evidence-based treatments (2013 ACC/AHA guidelines, class IIa). Biomarkers couldn’t be included in the MAGGIC project’s model, certainly because population was recruited from studies dating from 1980 to 2006, when natriuretic peptides were not available and treatments differ greatly, considerably limiting its applicability to contemporary patients. In contrast, our proSCANNED score appears more suitable to recent guidelines and current treatments. Furthermore, the multimarker approach could represent a promising tool combining markers involved in pathophysiology of HF and might in the future lead to other markers subjoin the mortality risk score^14^. For example ST2, member of the interleukin 1 receptor family, marker integrating inflammation, fibrosis and cardiac stress^16^ in combination with C-reactive protein were demonstrated as a valuable tool for identifying patients at risk of death^17^ and ST2 has been proposed as a new tool for management of patients with HF^18^. On the other side, biomarkers will never banish clinical predictors because clinical parameters remain robust but also because the benefit of HF therapy guided by brain natriuretic peptide is depending of patients’ comorbidities^19^. Likewise, future studies could reveal that newest treatments of heart failure have also a high predictive power. This would need and ad-hoc study including patients eligible to newest treatments.

There are other scores in the literature predicting survival in HF. The Seattle Heart Failure Model (SHFM)^6^ providing an estimation of 1-, 2- and 3-year survival while integrating large amunts of clinical (excluding diabetes), pharmalogical, device and laboratory characteristics, although this makes it complicated and poorly applicable to clinical practice. In addition, the Seattle Heart Failure Model and other previous studies focused on single cohort of patients with predominantly reduced EF^4,5^. Other studied variables can sound very interesting but not easily available such as: exercise tests, quality of life questionnaires assessed in HF-ACTION or less attractive like percentage of lymphocyte count in SEATTLE HF model^6^ or ApoA-1^14^. In opposition, the Cardiac and Comorbid Conditions HF (3C-HF)^20^ study aimed to predict all-cause 1-year mortality in HF patients with cardiac and comorbid variables and succeeded in balancing patients with preserved and reduced LVEF. Nevertheless 3C-HF model contains still a large number of variables and necessitated the use of electronic devices.

### Forces of the study

The proSCANNED score is tailored to clinical practice and applicable for all patients with HF. Therefore, only variables that are easily available are included in this score. We provide an external validation, which is the most challenging validation method, and which is rarely done. This score is able to predict 1-year survival with good discrimination and with an area under the curve in an external validation sample of 0.71 (95%CI : 0.63–0.79), demonstrating a good calibration and stability, compared to other scores^6,9,12,13,20,21^.

### Perspectives

Following guidelines improve outcomes^22^, but physicians need in clinical practice tools to better adjudicate times and means. Based on the proSCANNED score, various populations could be distinguished, guiding the management.

*1/the low-risk patients* with a score ≤ 2 have an expected < 5% predicted mortality at one year.

This could justify a level 1 follow-up (every 6 months by the cardiologist) with annual assessment of the natriuretic peptide and biology.

*2/the medium risk patients*—with a score of 3–4, have an 5–10% predicted mortality at one year.

*3/the high risk patients* with a score ≥ 5, which have a predicted mortality risk > 10% at one year. This could advocate for a strict follow-up with short-term reevaluation including frequent (monthly) consultation with the cardiologists, but also frequent biological or echography reevaluations.

### Limitations

The first limitation is due to the small sample size, limiting the generalization at this stage. Indeed, the MAGGIC study included 39,372 patients^7^ and the study which demonstrated the external validation included 51,043 patients from the Swedish Heart Failure Registry^8^. Nevertheless, this study uses predictors already well established in very large studies, and shows a discrimination and a calibration which may be sufficient to improve the management of patients. Second, the cohort is 10 years old, and we cannot take into account the new development in HF treatments. Third, patients with a high score were scarce, preventing from the generalization to patients with multiple risk factors. This could explain the low calibration in high scores. Finally, selection bias may arise from the complete-case analysis, and from the specialized setting where patients were recruited. All these limitations may decrease the applicability to current patients. This should be assessed using an ad-hoc prospective validation study with sufficient power. Nevertheless, these results show that a user-friendly and stable score can be derived.

Finally, some aspects could be mis-evaluated, especially the compliance of the patient, his preferences or environmental factors^23,24^.

## Conclusion

Estimating prognosis is a key element of HF management. The proSCANNED score appears as a new tool, easy to integrate in daily practice. However, it presents with limitations that may decrease its usefulness. Further studies will aim to validate and update the score on larger and more recent populations, and to assess the impact of this approach to improve the management of patients with HF.

## Supplementary Information

Supplementary Information

## Data Availability

Data are available on demand to the corresponding author.

## Abbreviations

ACE-I: Angiotensin converting enzyme inhibitor
AF: Atrial fibrillation
ARBs: Angiotensin II receptor antagonist
AUC: Area under the curve
BMI: Body mass index
CI: Confidence interval
CKD-EPI: Chronic Kidney Disease Epidemiology Collaboration
COPD: Chronic obstructive pulmonary disease
GFR: Glomerular filtration rate
HF: Heart failure
ICD: Implantable cardioverter-defibrillator
LVEF: Left-ventricular ejection fraction
MAGGIC: The Meta-Analysis Global Group in Chronic Heart Failure
MRA: Mineralocorticoid receptor antagonist
Nt-proBNP: Nt-pro-Brain Natriuretic Peptide
NYHA: New York Heart Association
P: P-value
SBP: Systolic blood pressure
